# ICESP, a research hub within a total cancer care center

**DOI:** 10.6061/clinics/2019/e1113

**Published:** 2019-01-25

**Authors:** Roger Chammas, Joyce Chacon Fernandes, Paulo Marcelo Gehm Hoff

**Affiliations:** Instituto do Cancer do Estado de Sao Paulo (ICESP), Hospital das Clinicas HCFMUSP, Faculdade de Medicina, Universidade de Sao Paulo, Sao Paulo/SP, BR

In its last special issue (vol 73, suppl 1), Clinics celebrates the 10th anniversary of the operation of Instituto do Câncer do Estado de São Paulo (ICESP). ICESP was established with the mission to provide total care for the patients with cancer who reside in São Paulo. ICESP is part of the Academic Health Center of the University of São Paulo, led by Faculdade de Medicina and Hospital das Clínicas da Faculdade de Medicina da Universidade de São Paulo. Under this arrangement, total cancer care also includes education, research and innovation. As a research center, ICESP has become a hub for cancer research in the state of São Paulo. This issue collects contributions of researchers working at ICESP and collaborators from other Brazilian institutions who have devoted their careers to the understanding of the complex set of diseases diagnosed as cancers.

The contributions reflect activities in the four large interconnected research programs developed at the institute: (a) Program of Cancer Prevention and Management, devoted to epidemiological studies that may contribute to the establishment of preventive programs, such as vaccination of virus-associated cancers and the organization of the health system to effectively provide cancer control for the population; (b) Program of Clinical Oncology, which runs a number of distinct cancer patient-centered projects, including clinical trials; (c) Program of Molecular Oncology, devoted to the understanding of cancer at the molecular level and the application of this knowledge for diagnosis and increasingly precise treatments; (d) Program of Innovative Cancer Diagnostics and Therapy, focused on the development of novel diagnostic or therapeutic strategies for cancer management, including the critical evaluation of implementation costs vs benefits/effectiveness of innovative medical technologies.

What we do for our patients at ICESP is not the only thing that matters. Rather, how we do it matters even more. We are certain that by creating an intellectually vibrant and productive atmosphere, we will be able to recruit the best health professionals to work for our patients. This issue celebrates this vision and anticipates the even more prolific years to come.

